# Interaction of Ethanolamine with Magnetite Through Molecular Dynamic Simulations

**DOI:** 10.3390/molecules30153197

**Published:** 2025-07-30

**Authors:** Nikoleta Ivanova, Vasil Karastoyanov, Iva Betova, Martin Bojinov

**Affiliations:** 1 Department of Physical Chemistry, University of Chemical Technology and Metallurgy, 8 Kliment Ohridski Blvd., 1756 Sofia, Bulgaria; n.ivanova@uctm.edu (N.I.); vasko_kar@uctm.edu (V.K.); martin@uctm.edu (M.B.); 2 Institute of Electrochemistry and Energy Systems, Bulgarian Academy of Sciences, 1113 Sofia, Bulgaria

**Keywords:** absorption, small molecules, atomistic MD, ClayFF

## Abstract

Magnetite (Fe_3_O_4_) provides a protective corrosion layer in the steam generators of nuclear power plants. The presence of monoethanolamine (MEA) in coolant water has a beneficial effect on corrosion processes. In that context, the adsorption of MEA and ethanol–ammonium cation on the {111} surface of magnetite was studied using the molecular dynamics (MD) method. A modified version of the mechanical force field (ClayFF) was used. The systems were simulated at different temperatures (423 K; 453 K; 503 K). Surface coverage data were obtained from adsorption simulations; the root-mean-square deviation (RMSD) of the target molecules were calculated, and their minimum distance to the magnetite surface was traced. The potential and adsorption energies of MEA were calculated as a function of temperature. It has been established that the interaction between MEA and magnetite is due to electrostatic phenomena and the adsorption rate increases with temperature. A comparison was made with existing experimental results and similar MD simulations.

## 1. Introduction

Monoethanolamine (MEA) is known to have an inhibiting effect on the corrosion processes of steam generators in power plants [1,2,3,4] and on low-alloy steels through adsorption on oxidized surfaces [5]. MEA is also a suitable agent for carbon capture and storage [6,7]. MEA exists in two main forms depending on pH, ethanolamine and ethanol–ammonium cation [7], with the molecule containing two different functional groups, OH and NH_2_ [6]. These properties of MEA make it an interesting target molecule for studying adsorption processes on metal oxides. One way to protect iron alloys from corrosion is to form an oxide layer [8]. Magnetite Fe_3_O_4_ is found to be the main protective layer on carbon and low-alloy steels. Its crystal lattice is typical of reverse spinel and the structure contains octahedral and tetrahedral–octahedral layers [9].

Density functional theory (DFT) calculations have been performed on the {111} Fe_3_O_4_ surface considering the stabilities for iron ions [10] and oxygen ions [11]. Other surfaces of magnetite have also been examined with DFT, and it has been found that the {111} surface is probably the most exposed in the crystals [12]. Good practices for DFT modeling of magnetite above the Verwey temperature are also discussed in detail [13]. DFT parameters are proposed for a description of water adsorption on the {001} surface of magnetite [14], and the adsorption energy of water on the Fe_3_O_4_ {111} has also been calculated [15].

A powerful tool for better understanding a range of phenomena, and, in particular, adsorption processes, is the molecular dynamics (MD) method. The accuracy of this computational technique depends on the correctness of the mechanical force fields imposed on the model systems. It is important that the field contains the correct parameters for electrostatic interactions [16]. For a system containing magnetite, it is necessary to modify the existing fields due to the complexity of the structure [17]. ClayFF is a field that perform well [18] with reliable parameters for surfaces in aqueous solutions. There are different modifications of the force field for atomistic description of the bulk and surface of the material [19,20].

In a previous study of ours [21], a model of the {111} plane of magnetite Fe_3_O_4_ was constructed. The electrostatic parameters were taken from an imposed field ClayFF [18] and a previously used methodology for magnetite in an aqueous environment [20]. The stability of the model was verified, and several properties of the crystal structure were investigated. Ammonia adsorption on that system was modeled at different temperatures. The root-mean-square deviation (RMSD) profile and radial distribution functions (RDF) were calculated, and an analysis of ammonia movement was provided. This system and its imposed parameters were determined as suitable for further consideration of adsorption processes.

MD simulations have shown that MEA forms both intermolecular and intramolecular hydrogen bonds in an aqueous environment, leading to the stabilization of the molecule [22]. In a further study, using the method of neglecting differential overlap (MNDO), the structures of both forms ethanolamine and ethanol–ammonium cation were calculated, and suitable isomers were proposed for the present study [23].

These two works serve as a good basis for the study of ethanolamine in the presence of water and magnetite. The main goal of the current research is to examine the possibility of adsorption of the two existing forms of ethanolamine, the molecule and the ethanol–ammonium cation, on a surface under conditions as close as possible to the steam generator operation, as model systems are at high pressures, such as 90 bar. Of interest for the study are three target temperatures (423 K; 453 K; 503 K), within the working range of secondary nuclear plant circuits and already studied experimentally [23]. Another important task of the present work is to show whether ethanolamine adsorbs with the nitrogen atom to the oxide surfaces. Such a mode of adsorption leads to compensation of the oxygen charges from the surface, and ethanolamine has an inhibiting effect on corrosion phenomena.

## 2. Results and Discussion

**Figure 1:**
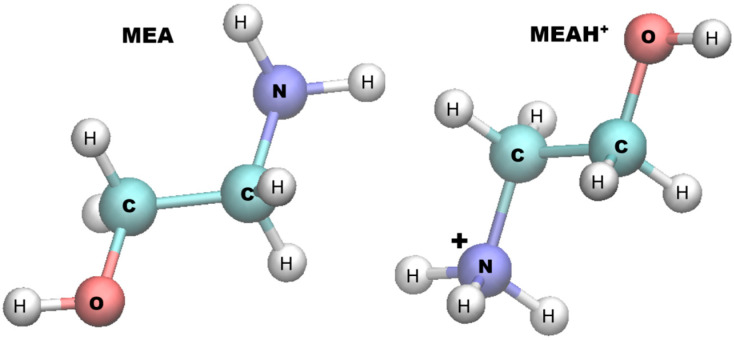
Molecular structure of ethanolamine (MEA) and ethanol–ammonium cation (MEAH^+^).

The behavior of two forms of MEA (ethanolamine and ethanol–ammonium cation, Figure 1) in aqueous medium was monitored in the vicinity of a magnetite {111} surface. Detailed information on the composition, size, and validation of the model system is presented in Section 3.

The analyses were performed on the trajectories with the coordinates and velocities of each atom from the atomistic molecular dynamic simulations. Depending on the type of parameters considered for the two systems, different parts of the trajectory were used. The potential and adsorption energies of MEA were calculated as a function of temperature. RMSD profiles were obtained for both forms in the entire time interval. The average distance to the magnetite surface was determined and an average adsorption rate was derived. It was established which groups participate in the interaction and radial distribution functions profiles were calculated for them. The results are commented on and compared with molecular dynamics simulations of similar systems and experimental data where possible.

### 2.1. Adsorption Energy

**Figure 2:**
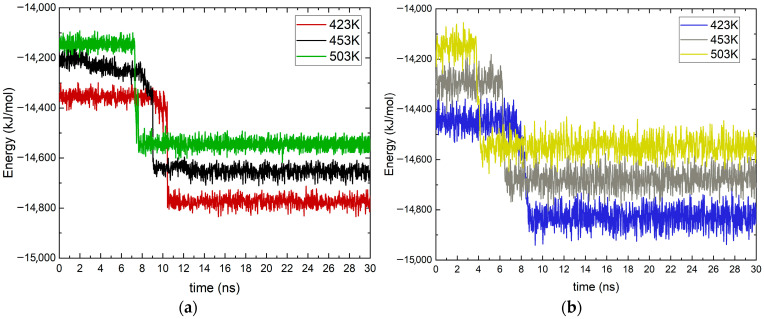
Potential energy of the molecules considered versus simulation duration in ns at different temperatures in K (marked with different colors): (**a**) ethanolamine; (**b**) ethanol–ammonium cation.

The potential energy component of intramolecular contributions for both forms of MEA was calculated over the entire time interval. Energy groups of the atoms contained in the molecules were assigned using the Large-Scale Atomic/Molecular Massively Parallel Simulator (LAMMPS), and the energy between each two atoms was determined. The calculation was averaged for six molecules in the system. The profiles were identical for the three parallel simulations at the studied temperatures. Frames were recorded every picosecond, and the obtained data are presented in Figure 2.

The results show that the potential energy profiles for the two systems are similar, and a dependence on temperature change has been reported.

There is a large number of fluctuations in the calculated graphs, but despite those specific trends, the behavior of the molecules of interest can be clearly identified. The system containing the cation exhibits more fluctuations than the uncharged molecule. This suggests that this system, although it has reached equilibrium, is the more unstable of the two.

In both systems, there is a sharp decrease in potential energy, which is an indication of adsorption on the magnetite surface. The change in the profile occurs at similar times for both forms of MEA, but for the cation, the process is faster. At a temperature of 423 K for ethanolamine, the change in energy occurs at about 10 ns, while for the ethanol–ammonium cation is at 8 ns. This acceleration of the charged molecule is also visible at the two higher temperatures. The process of the cation is visually fastest at 503 K (4 ns), and in this case, the decrease in energy is also sharp. For the neutral form of MEA, a more subtle change is also observed at 503 K, since at lower temperatures, there is a slight drop in the potential energy profile. This suggests that the adsorption mode differs at the three studied temperatures. Similar behavior with a decrease in potential energy in the probable moment of adsorption has been reported in other molecular dynamics simulations [24].

A clear dependence of potential energy on temperature is observed. In both systems, there is an increase in the profiles both before and after probable adsorption processes. This is also typical for molecular dynamics simulations since potential energy is a component of the total energy of the systems.

Obtaining the entire energy profile of the studied systems allows us to estimate the adsorption energy of both forms of MEA on the Fe_3_O_4_ surface. Calculations were performed on a portion (5 ns) of the tractors at different times at which supposed adsorption occurred. The energy was calculated by Equation (1) [24] and averaged over the parallel simulations.

(1) $$E_{ads}=E_{tot}-E_{slab/w}-E_{mol}$$

In this equation, *E_tot_* is the total energy of the system, *E_slab/w_* is the energy of the crystal slab in the aqueous medium, and *E_mol_* is the energy of all the MEA molecules before adsorption.

The calculation of the energy contributions critically depends on the imposed force field (FF) for Fe_3_O_4_ in the MD simulations [25]. In the current development, a modified version of ClayFF has been implemented. The comparison of the data is made only as trends, but not as absolute values. Since the presented adsorption energy depends on the number of molecules adsorbed to the surface, it is worth noting that in the studied systems, all six particles are adsorbed. The data are presented in Table 1.

The approach to calculating the adsorption energy in the current research does not distinguish between tetrahedral and octahedral iron ions, as well as the presence of oxygen ions but is applied to the entire material. For both MEA molecule and cation model systems, an increase in adsorption energy with temperature has been reported. Despite the increase, there is no change in the sign of the energy obtained and no desorption has been observed within the simulation time. Adsorption energies in a similar range were obtained for water molecules on the Fe_3_O_4_ {001} surface [26]. The same method was used to obtain the adsorption energies of phenol, p-chlorophenol, and p-nitrophenol on a magnetite surface [27]. The adsorption energies for NOx molecules on the γ-Fe_2_O_3_ {111} surface were also studied with emphasis on the site of adsorption [28].

From the potential energy profiles (Figure 1) after the adsorption moment, a decrease is seen in the system containing the ethanol–ammonium cation. This decrease is also reflected in the adsorption energy. Another possible reason for the lower energy is the electrostatic interaction between the charged parts of the cation and the oxygen ions on the magnetite surface. It is noted that for the ion, the increase in energy with temperature is greater than for MEA. This may be due to the increasing instability of the cationic form at high temperatures. To analyze the state of the ethanol amine molecules in more detail, the root-mean-square deviation profiles (RMSD) was calculated.

### 2.2. Ethanolamine Stability

**Figure 3:**
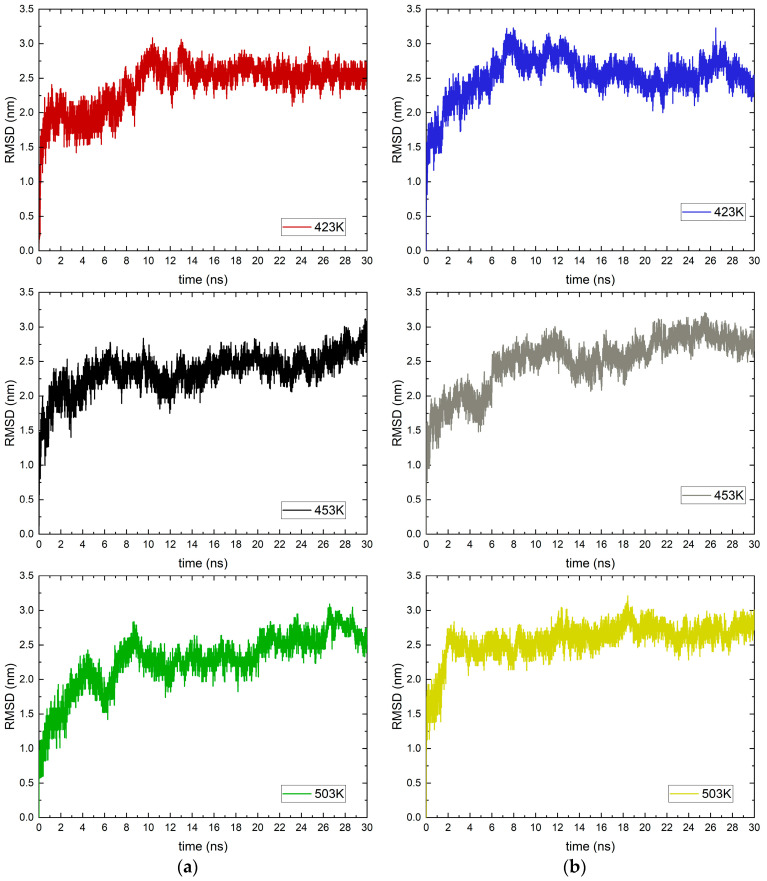
Root-mean-square deviation (RMSD) at different temperatures in K (marked with different colors) versus simulation duration in ns of (**a**) ethanolamine; (**b**) ethanol–ammonium cation.

The root-mean-square deviation (RMSD) profiles were calculated for the entire time interval of the simulations. The data are averaged for the six MEA molecules in both forms. The frames for the calculation are 0.5 ps, which allows for detailed analysis. Due to the frequent collection of velocities and positions of atoms, the parameter is tracked only for one of the three parallel simulations in which the best interaction is reported. The data at all the temperatures studied are shown in Figure 3.

In both studied systems, there is a change in the configuration of the adsorbed molecule in the part of the trajectory corresponding to the adsorption at 423 K. In MEA, there is a decrease in fluctuations after the adsorption occurs, which suggests the stability of the obtained configuration and the low probability of desorption. The situation is similar with the cation, although the decrease in fluctuations, and of the entire profile in general, is only noticeable at the end of the trajectories (after 27 ns). The profile of the neutral form of MEA at 453 K does not give a clear indication of the adsorption time, but a slight increase is observed around 7 ns. In contrast, for the cation there is a clear change in the height of the RMSD at 6ns, after which the profile somewhat repeats the stability at a temperature of 423 K. For the cation, there is a clear increase in the RMSD of about 4 ns at a temperature of 503 K. In this system, an increase in fluctuations is also observed. Although a slightly faster adsorption is reported here compared to the other systems, the presence of instability in the system could lead to desorption at longer simulation times. At the same temperature, the neutral form of MEA experiences serious conformational changes in the structure of the molecule. This could be attributed to the search for a suitable configuration of the molecule relative to the surface of the metal oxide. In general, the profiles after adsorption do not show specific instabilities, but there are general increases in height, and for the cation there is also an increase in fluctuations.

The energy profile and the RMSD profile do not provide accurate information about the moment when ethanolamine and ethanol–ammonium cation adsorb on the magnetite surface. Therefore, the movement of target molecules within the simulations was calculated.

### 2.3. Kinetics of Ethanolamine Adsorption

**Figure 4:**
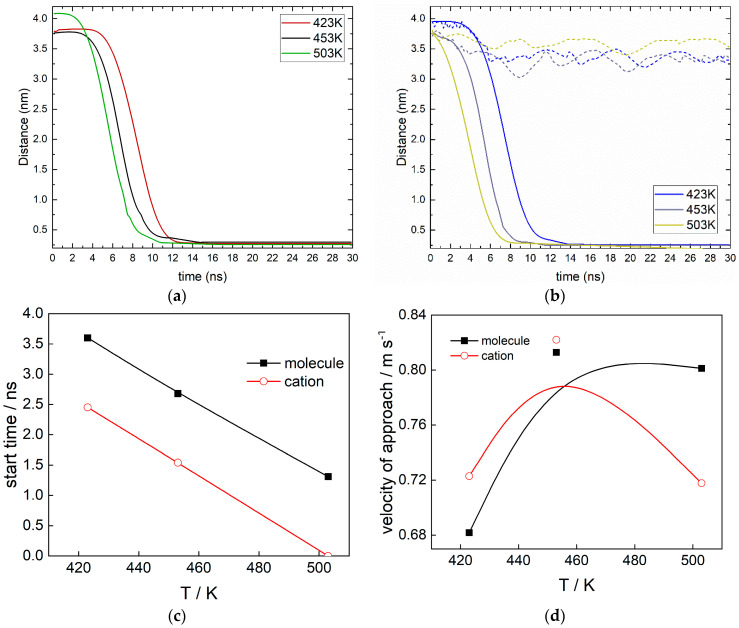
The distance from the molecules: (**a**) ethanolamine; (**b**) ethanol–ammonium cation to the surface of magnetite at the temperatures studied marked with different colors (K) as a function of simulation time (ns), dashed lines correspond to hydroxyl ions; (**c**) start time of approach and (**d**) velocity of approach estimated from the distance–time curves as a function of temperature.

The average distances between the center of mass of the two molecules of interest, as well as hydroxyl ions, and the oxide surface were calculated. Averaging was performed for the six particles in the model systems in the three parallel simulations, which results in a smooth profile, although the motion is not uniform. The final form of the calculations is shown in Figure 4 for the three temperatures studied. The tracking of the motion of ethanolamine and ethanol–ammonium cation towards the magnetite surface was considered for the entire duration of the trajectories.

The initial coordinates of the target molecules are identical for all temperatures. They are placed far from the surface randomly along the *xy* plane. After heating the systems to the respective temperature, there is a change in the position of the molecules for both forms of MEA. This is reflected in the different initial distance of the productive part of the trajectories in Figure 4. Despite the different starting positions, it becomes clear that in the systems considered, the adsorption kinetics depends on the temperature and charge of the particle. In general, the cation moves slightly faster than the neutral molecule.

In the system containing the neutral form of MEA, the adsorption rate increases with temperature. This is expressed as a decrease in both the starting time of the process, and the time for all molecules to reach the surface, as well as the calculated velocity of approach (Figure 4c–d). The approach is the fastest at 503 K, although in that case, the average particle distance is greater than at lower temperatures. At this temperature, the adsorption process of ethanolamine ends after 10 ns. At 423 K, the ethanol–ammonium cation approach process begins after 4 ns and ends after 15 ns. With increasing temperature, this process accelerates, and at 503 K the adsorption process starts at the beginning of the simulation. The reason for the faster movement of the cation is its charge in the NH_3_^+^ group, which seeks to reach the negatively charged oxygen ions of the magnetite. At this temperature, all ethanol–ammonium cations have completely reached the magnetite after 8 ns. At 453 K, the situation is similar, but the process itself begins later. The time for which the particles are adsorbed is >9 ns. Since the differences in the adsorption time are small, it can be concluded that at higher temperatures, electrostatic interactions prevail. The average rate of the slowest process (ethanolamine at 423 K) is 0.39 nm per ns; for the fastest (ethanol–ammonium cation at 503 K), it is 0.46 nm per ns.

In the system containing the cationic form, practically no approach of hydroxyl ions was observed (Figure 4b). In the initial moments of the MEA approach, the OH ions experience the effect of the starting adsorption. There is a certain decrease in the average distance to the magnetite surface. Within the computational time, the hydroxyl ions remain in the bulk water and no definite approach of a given particle to the surface is observed. Further analysis of the behavior of the OH ions will not be considered.

Similar profiles have already been observed in our previous study [21]. Such behavior was also reported for the adsorption of ethanol on the Al {111} surface [24].

The suggested absorption rate from the calculated distances is an average rate type; thus, it does not provide a complete picture of the adsorption kinetics and mechanism. Additional important information on the adsorption mechanism can be obtained by determining which parts of the ethanolamine molecule are involved in the interaction with the magnetite surface.

### 2.4. Surface Interaction

**Figure 5:**
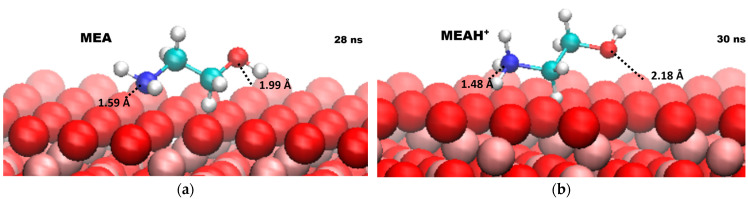
Average distances between functional groups and the magnetite surface: (**a**) ethanolamine (MEA); (**b**) ethanol–ammonium cation (MEAH^+^).

The average distances between magnetite and HO and NH_3_^+^ groups were calculated. The analysis was performed on the last 3 ns of the trajectory after the final approach of the target molecules to the surface occurred. The data averaged over three parallel simulations for the temperatures studied are shown in Table 2 and Figure 5.

The values obtained for both groups are identical at different temperatures. This shows, once again, that electrostatic interactions have a greater contribution than the temperature after the process is completed. Also, despite the reported instabilities in the systems at high temperatures, similar distances indicate a low probability of ethanolamine desorption.

For both forms of MEA, the nitrogen atom is located closer to the magnetite surface than the oxygen atom. In the adsorption process, the NH_x_ group participates more significantly and has a key role in the mechanism. It is striking that in the charged form (ethanol–ammonium cation) the nitrogen atom is closer than in the uncharged form. Also, in this configuration, the oxygen atom is further away from magnetite relative to the neutral molecule. This is another indication that electrostatic forces make a significant contribution to the adsorption process. Distances in a similar range have been observed for different isomers of MEA on hydroxylated Cr_2_O_3_ [29]. The data correspond well with those obtained in the present study, despite the different structure of the oxide.

**Figure 6:**
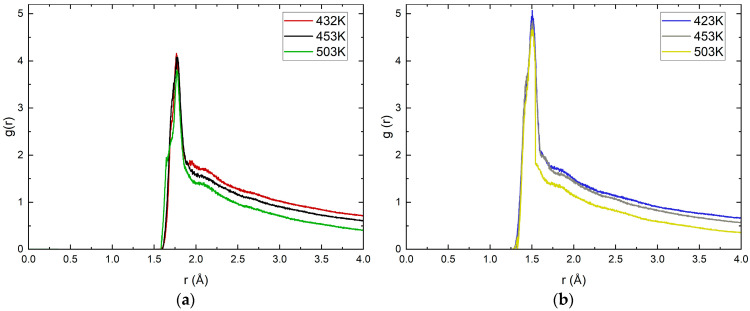
Radial distribution functions (RDFs) at different temperatures in K (marked with different colors) of (**a**) ethanolamine and (**b**) ethanol–ammonium cation.

Additional information about the interaction between the NH_x_ group and magnetite can be obtained from the radial distribution functions (RDFs) between the oxygen atom from the oxide surface and the nitrogen atom from the adsorbed molecule. Figure 6 presents the RDFs for both forms of ethanolamine from the last 3 ns of the calculated trajectories.

The RDF data largely confirm the conclusions drawn from the calculated average distances. In both systems there is a clearly pronounced peak, i.e., there is a clearly preferred distance between the atoms under consideration. The intensity of the peak in the ethanol–ammonium cation is expectedly greater than that in the neutral molecule. This is, again, an indication that in this form, the electrostatic forces are determining.

The temperature dependence in both forms of ethanolamine is small. A characteristic decrease in the intensity of the peak with increasing temperature is observed for RDF profiles. For the neutral form of MEA at 503 K there is a broadening of the peak which may correspond to the reported significant changes in the RMSD profile (Figure 3). The changes in the configuration of the two forms of MEA reported by RMSD do not lead to significant differences in the RDF profiles. The most likely distances between the oxygen atoms of magnetite and the target group are weakly affected by temperature, which is evidence for a dominant electrostatic contribution to the interaction. The data for the radial distribution functions and the observed trends with temperature are similar to those obtained from MD simulations for ethanol adsorption on aluminum [30].

The analyses performed do not consider the specific location of the magnetite surface on which the adsorption processes occur. It was found that the preferred site for adsorption is towards oxygen atoms without emphasis on the type of iron atom (octahedral or tetrahedral) since the electrostatic contribution is significant. Also, the calculated adsorption rate does not provide accurate information about the possible kinetic model. These are important components of the analysis, and present good opportunities for future studies of this type of adsorption process.

### 2.5. Comparison with In Situ Electrochemical Data

**Figure 7:**
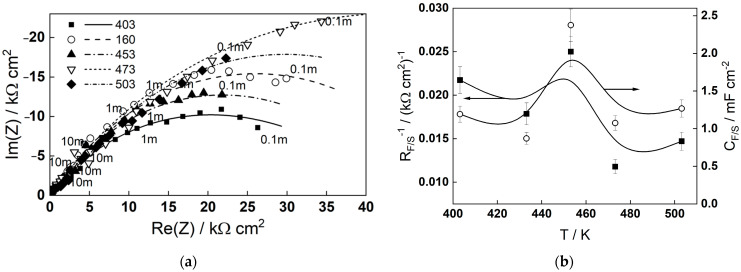
(**a**) Dependence of the impedance spectra of low-alloyed steel (0.6%Cr) on temperature after 72 h of exposure in 0.1 mmol dm^−3^ MEA solution. Points—experimental data, lines—best-fit calculation according to the mixed-conduction model. Parameter is frequency in Hz; (**b**) temperature dependence of the inverse of the charge transfer resistance (R_F/S_^−1^) and the interfacial capacitance (C_F/S_) on temperature.

In a companion paper [23], the corrosion of low-alloyed steel (0.6%Cr) in MEA solution was studied via in situ electrochemical impedance spectroscopy. The dependence of impedance spectra on temperature after 72 h of exposure to a 0.1 mmol dm^−3^ MEA solution is shown in Figure 7. The dependence of the impedance at low frequencies (0.1 mHz), identified with the polarization resistance, i.e., the inverse of the corrosion rate, is non-monotonous and passes through a minimum at 453 K. Whereas the complete interpretation of the spectra was presented in [17], in this study, we focus on the interfacial parameters estimated from the spectra—the inverse of the charge transfer resistance (R_F/S_^−1^) associated with the interfacial reaction rate, and the interfacial capacitance (C_F/S_) related to adsorption at the oxide/solution interface. Both parameters pass through a maximum at 453 K, which correlates well with the acceleration of adsorption kinetics of MEA between 453 and 473 K, as indicated by the molecular dynamic simulations. This, in turn, could lead to a more efficient adsorption of MEA at higher temperatures, and accordingly to a lower corrosion rate of the low-alloyed steel, in accordance with what was found experimentally [23].

The comparison to electrochemical data is only qualitative at this moment, since the main goal of our previous paper [23] was to estimate the role of MEA in the oxidation and corrosion release processes of low-alloyed steel. Further investigations with a broader range of potentials are needed to estimate adsorption energy from capacitance and compare that with MD simulation results. Such measurements will be attempted in the future.

## 3. Materials and Methods

In the present study, magnetite spinel (Fe_3_O_4_) was studied using a high-pressure structure [31]. A detailed analysis of the structure and stability of the model structure used was described in a previous study [21]. The ethanolamine parameters have already been validated for several types of isomers [22].

### 3.1. Materials

**Figure 8:**
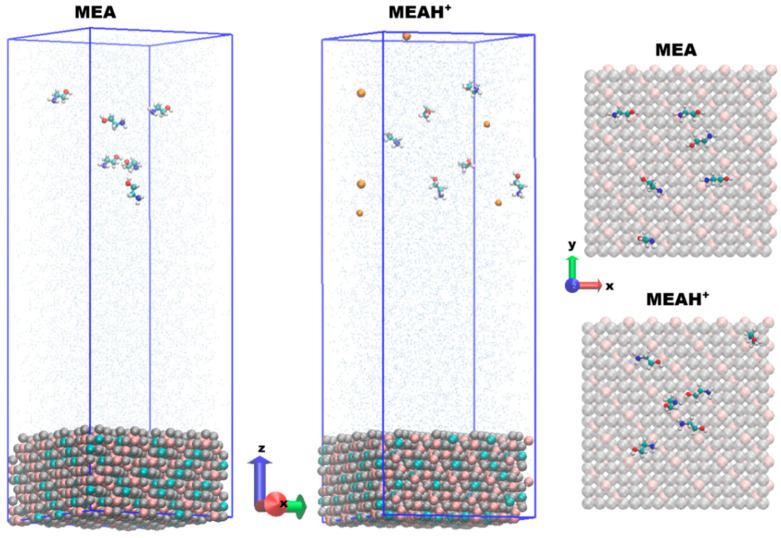
Initial coordinates (side view) and final coordinates (top view) of the modular systems at 503 K; hydroxyl ions are shown in yellow.

The studied material contains 48 unit cells (2688 atoms), resembling the {111} plane with a thickness of 2.5 nm. In the current development, a relaxed configuration of the magnetite layer at 293 K was used. Periodic boundary conditions (PBCs) with a size of 10 nm were imposed. It was found that the presence of over 3437 water molecules reproduces the density of the medium well at this temperature. Ethanolamine has different isomeric structures, but two preferred ones were selected from the DFT calculation [23]. Two model systems were considered, with six molecules of ethanolamine and ethanol–ammonium cation added to this structure. To ensure a neutral charge of the ethanol–ammonium cation system, six hydroxyl anions were added. Due to overlapping atoms, specific water molecules were deleted from the systems and new ones were added. Thus, the model with the molecular form of MEA contains 3421 molecules, and that with the cation contains 3416 molecules. The structures are visualized in Figure 8.

### 3.2. Methods

With the two model systems thus constructed, atomistic molecular dynamics simulations were carried out at the following three temperatures: 423 K; 453 K; and 503 K. The LAMMPS 2Aug2023 (Large-Scale Atomic/Molecular Massively Parallel Simulator) software package suitable for crystal structures was used for MD calculations. The imposed force field is ClayFF with a modification for hematite–water interaction [32], which provides good results for the type of systems [21]. The missing parameters for ethanolamine validated for the liquid phase [33] were added to the field. The water model used is TIP3P [34].

**Figure 9:**
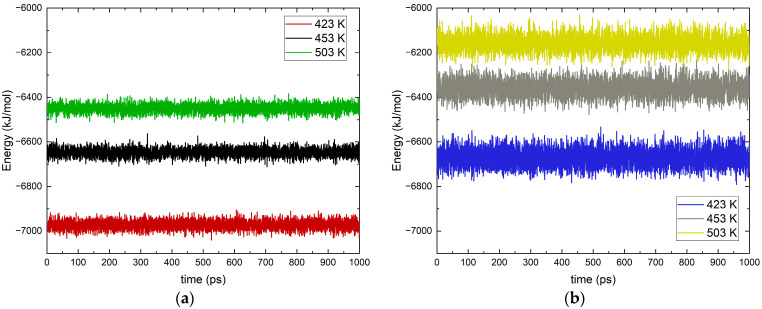
Total energy in the systems: (**a**) ethanolamine and (**b**) ethanol–ammonium cation at different temperatures in K (marked with different colors).

With the constructed systems, only the added ethanolamine and ethanol–ammonium cation molecules were minimized since the remaining system was relaxed. After reaching a minimum in potential energy, the two model systems were heated to the selected temperatures. The heating of all six systems was performed in the NVT ensemble with Nose–Hoover’s thermostat [35,36], with the scaling factor 1 ps for the ethanolamine and 0.5 ps for the ethanol–ammonium cation. For only the cation at 503 K, the was scaling factor 0.1 ps, which makes the heating stage significantly longer (about 5 ns). After reaching the target temperatures, the systems established equilibrium levels of the total energy in the systems, as shown in Figure 9.

Despite the increase in energy with temperature, the average values remain constant over time. This provides grounds for conducting productive simulations in the NPT ensemble with a pressure of 90 bar. The integration time step is 1 fs and the trajectory data are recorded at 0.5 ps. During the productive simulations, the thermostat and barostat are not changed and the electrostatic interactions are accounted for with particle–particle–particle–mesh (PPPM) with a precision factor of 1 × 10^−6^, and the Lennard–Jones potential interactions cut off is 12 Å.

The duration of the productive MD simulations is 30 ns and the analyses were performed on different parts of the trajectories depending on their type. The final configurations are presented in Figure 8. For the visualization of the systems, the VMD 1.9.4a (Visual Molecular Dynamics) package was used [37].

## 4. Conclusions

In the present study, the adsorption of ethanolamine and ethanol–ammonium cation to the magnetite {111} surface was monitored using the molecular dynamics method. The constructed systems are based on previous ones and are placed at three different temperatures (423 K; 453 K; 503 K), operating for steam generators in nuclear power plants. In the systems considered, the potential energy for both forms of ethanolamine and adsorption energy were obtained. The stability of the adsorbed molecules was described by root-mean-square deviation, and the approach of the molecules to the magnetite surface was considered by the average distance. The surface interaction was estimated with average distance between the functional groups of ethanolamine and radial distribution functions.

The considered properties (except the average distance of the functional groups) demonstrate a clear dependence on temperature. The increase in temperature leads to faster adsorption, but causes certain instabilities in the systems, as no desorption was visually observed within the simulation times. During the interaction of both molecule forms with magnetite, the nitrogen atom is more actively involved. In some systems, a predominance of electrostatic interactions over the effect of temperature is observed (such as for the ethanol–ammonium cation at 503 K). The results obtained correlate well with data from similar molecular dynamic systems, and a qualitative comparison with available electrochemical experiments was performed.

## Figures and Tables

**Table 1:** Adsorption energy for both forms of ethanolamine at the studied temperatures.

Temperature, K	Ethanolamine, kJ·mol^−1^	Ethanol–Ammonium Cation, kJ·mol^−1^
423	−179.506	−184.078
453	−153.686	−156.824
503	−139.217	−141.483

**Table 2:** Average distance between the functional groups of ethanolamine and the magnetite surface for the studied temperatures.

Temperature, K	Ethanolamine, Å		Ethanol–Ammonium Cation, Å	
	*OH*	*NH_2_*	*OH*	*NH_3_^+^*
423	1.96	1.63	2.11	1.46
453	1.94	1.62	2.15	1.48
503	1.97	1.69	2.14	1.47

## Data Availability

The data presented in this study are available on request from the corresponding author due to privacy reasons.
